# A Quantified Risk-Scoring System for the Recurrence of Meningiomas: Results From a Retrospective Study of 392 Patients

**DOI:** 10.3389/fonc.2020.585313

**Published:** 2020-10-05

**Authors:** Zhangzhang Zhu, Chengde Wang, Jiadong Xu, Chunyong Wang, Lei Xia, Qun Li, Jianglong Lu, Lin Cai, Weiming Zheng, Zhipeng Su

**Affiliations:** ^1^Department of Neurosurgery, First Affiliated Hospital of Wenzhou Medical University, Wenzhou, China; ^2^Department of General Surgery, Zhuji People's Hospital of Zhejiang Province, Shaoxing, China; ^3^Department of Neurosurgery, Wencheng County People's Hospital, Wenzhou, China

**Keywords:** meningioma, recurrence, prognosis, risk model, scoring system

## Abstract

**Aim:** This study aimed to identify the independent risk factors of recurrence in patients undergoing primary resection of meningioma and construct a scoring system for the prediction of the risk of postoperative recurrence.

**Materials and Methods:** The clinical data of 591 patients who underwent primary surgical resection for meningioma at the First Affiliated Hospital of Wenzhou Medical University between November 2010 and December 2016 were retrospectively reviewed. The clinical, radiological, and pathological characteristics were evaluated, and the independent risk factors for recurrence were identified via receiver operating characteristic (ROC) curve and logistic analyses. A scoring system that included these independent risk factors was used to construct a risk-predicting model that was evaluated via a ROC curve analysis. The recurrences of different subgroups were observed by Kaplan-Meier's curves.

**Results:** The clinical data of 392 patients with meningioma were used to construct the scoring system. The logistic analysis showed that sex (OR = 2.793, 95% CI = 1.076–7.249, *P* = 0.035), heterogeneous tumor enhancement (OR = 4.452, 95% CI = 1.714–11.559, *P* = 0.002), brain invasion (OR = 2.650, 95% CI = 1.043–6.733, *P* = 0.041), Simpson's removal grade (OR = 5.139, 95% CI = 1.355–19.489, *P* = 0.016), and pathological grade (OR = 3.282, 95% CI = 1.123–9.595, *P* = 0.030) were independent risk factors for recurrence. A scoring system was developed and used to divide the patients into the following four subgroups: subgroup 1 with scores of 0–75 (*n* = 249), subgroup 2 with scores of 76–154 (*n* = 88), subgroup 3 with scores of 155–215 (*n* = 46), and subgroup 4 with scores of 216–275 (*n* = 9). The incidences of recurrence in each subgroup were as follows: subgroup 1, 1.2%; subgroup 2, 5.7%; subgroup 3, 26.1%; and subgroup 4, 66.7% (*P* < 0.001). The scoring system reliably predicted the postoperative recurrence of meningioma with a high area under the ROC curve.

**Conclusions:** Our scoring system is a simple and reliable instrument for identifying meningioma patients at risk of postoperative recurrence and could help in optimizing individualized clinical treatment.

## Introduction

Meningioma is the most prevalent primary intracranial tumor and accounts for ~15–30% of all primary intracranial neoplasms (1). According to the current 2007 World Health Organization (WHO) classification system, most meningiomas are benign (~80%). The WHO classification distinguishes three histological grades (I-II-III) and 15 subtypes (2). Currently, the method most commonly used in the clinic to predict recurrence is risk stratification based on the WHO grade as the tumor histological grade has been demonstrated to predict the postoperative risk of recurrence following treatment (3, 4). Some studies have also reported other risk-related factors for recurrence, including radiological characteristics, age and Simpson's removal grade (5, 6). However, despite current studies investigating the prognosis of meningioma, a reliable prediction system is still lacking (7, 8). Therefore, we retrospectively analyzed data obtained from patients with WHO grades I, II, or III meningiomas who were treated in our hospital. The aims of this study were to identify the prognostic factors that influenced the postoperative recurrence of tumors, construct a new scoring system and risk-rating model for the prediction of postoperative recurrence, and support the optimization of treatment strategies for patients.

## Materials and Methods

### Patients and Data

**Figure 1:**
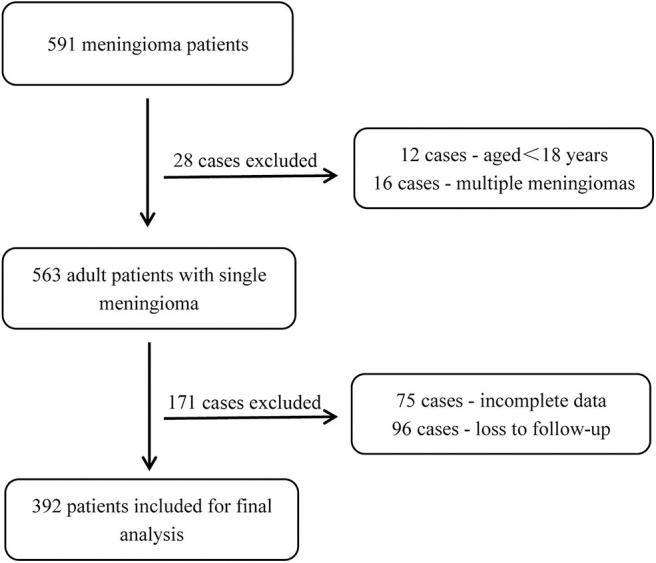
Flowchart of the patient selection process.

Patients with meningioma who underwent primary surgical treatment at the Department of Neurosurgery at the First Affiliated Hospital of Wenzhou Medical University between November 2010 and December 2016 were enrolled in this retrospective study. Among all 591 patients enrolled in our study, the demographic and clinical data were retrospectively collected using all available inpatient and outpatient reports and records. The follow-up period was up to October 2019 or the first recurrence after primary tumor resection. The inclusion criteria were as follows: (1) an age ≥18 years; (2) available preoperative and postoperative magnetic resonance imaging (MRI) (including T1-weighted, T2-weighted, and contrast-enhanced T1-weighted) data; (3) pathologically confirmed meningioma based on the WHO histological grading system; and (4) complete postoperative follow-up data. The exclusion criteria included a lack of complete imaging data, loss to follow-up, and multiple meningiomas. Based on these criteria, 392 of the 591 patients were included in the study analyses (Figure 1). This study was approved by the Clinical Research Ethics Committee of the First Affiliated Hospital of Wenzhou Medical University. Given the retrospective nature of the study, patient informed consent was waived.

### Recorded Variables

We collected the patients' clinical, radiological, and pathological characteristics, including sex, age, preoperative Karnofsky performance scale (KPS), tumor location, tumor size, tumor shape, peritumoral edema, dural tail sign size, tumor calcification, tumor-surrounding vessels, tumor basal size, heterogeneous tumor enhancement, tumor–cortex interface, Simpson's removal grade, pathological grade, brain invasion, and Ki-67 index.

In our study, a regular tumor shape was defined according to MRI as round or oval, and an irregular tumor shape included fusiform and other irregular shapes. Peritumoral edema was evaluated based on T2 predominant sequences obtained in MR screenings. The edema–tumor volume ratio was defined according to the Edema Index (EI). We estimated the tumor and edema volume based on the results of the MR scan as follows: the maximum perpendicular diameters were measured on axial images, and the extent in the coronal direction was estimated as the number of axial images that displayed the structure multiplied by the slice thickness. The relationship between peritumor brain edema (PTBE) and the tumor volume was defined as EI = (V_Edema_+V_Tumor_)/V_Tumor_; when there was no edema, the result was 1 (9, 10). T2 predominant MR screenings were used to examine the tumor-cortex interface, which was classified as follows: (1) marked interspace when there was a distinct interval (>1 mm wide) between at least 50% of the tumor and the surrounding cortical surface; (2) a regular border when there were no gaps or irregular boundaries between the tumor and the subcortical surface, but regular boundaries were observed across more than 50% of the surface; and (3) an irregular border when there was no clear cortical contour on more than 50% of the surface of the tumor (11, 12). The tumor-surrounding vessels were defined based on a T2-weighted image in which the sign of empty blood vessels surrounded the tumor blood vessel. Heterogeneous tumor enhancement was defined based on T1-weighted contrast images when the tumors were enhanced inhomogenously, and no apparent hyperintensity in the part of the tumor-involved area was observed on a postcontrast T1-weighted image. The tumor size, dural tail sign size and tumor basal size were measured on T1-weighted contrast images.

Tumor recurrence was defined as the formation of a new contrast-enhanced nodule in the previous resection cavity, the formation of a 95% isodose line or residual tumor progression in patients who underwent subtotal resection. During the follow-up period, relapse was assessed by a senior neurosurgeon and an experienced neuroimaging specialist.

### Statistical Analysis

The Kolmogorov-Smirnov test was used to evaluate the distribution uniformity of the continuous parameters. The normally distributed data are expressed as the mean ± standard deviation, and the non-normally distributed data are expressed as the median and interquartile range (IQR). An independent *t*-test and Mann-Whitney *U*-test were used to analyze the differences between the groups in the continuous variables, while the chi-square test and Fisher's exact test were used to analyze the differences between the groups in the categorical variables. We plotted receiver operating characteristic (ROC) curves and selected the maximal value of the Youden index as the cutoff point for the tumor size, tumor basal size and total risk scores. The patients were grouped according to this cutoff point. Based on the univariate analysis, a multiple logistic regression analysis was conducted to calculate the odds ratio (OR) and 95% confidence interval (CI) of the independent variables. The area under the ROC curve (AUC) was used to evaluate the accuracy of our scoring system based on the clinicopathological characteristics of the individuals. Kaplan-Meier's curves with a log-rank test were performed to observe the recurrence of meningioma in different subgroups. Statistical significance was indicated by *P* < 0.05. The statistical analyses were carried out using SPSS software (SPSS 22.0 Inc., Chicago, IL).

## Results

### Patient Characteristics

The clinical, radiological and pathological data of 392 meningioma patients were systematically reviewed. The median follow-up duration was 60 months (IQR = 46–78 months). The baseline characteristics are shown in Table 1. The majority of patients were female (*n* = 268, 68.4%), and the median age of all patients was 55 years (IQR = 47–63 years). The tumor sites included the cranial convexity (167, 42.6%), skull base (122, 31.1%), parasagittal sinus (51, 13.0%), and other locations (52, 13.3%). The pathological grades included WHO grade I (362, 92.4%), WHO grade II (26, 6.6%), and WHO grade III (4, 1%) meningioma. Of the 392 patients included in the analyses, 26 cases (6.6%) experienced recurrence after surgery.

**Table 1:** Clinical characteristics.

**Characteristic**	**Value (*N* = 392)**
Sex	
Female	268 (68.4%)
Male	124 (31.6%)
Age (y)	55 (47-63)^*^
Preoperative KPS	
>70	345 (88.0%)
≤70	47 (12.0%)
Tumor location	
Convexity	167 (42.6%)
Skull base	122 (31.1%)
Parasagittal sinus	51 (13.0%)
Other	52 (13.3%)
Tumor size (mm)	
>42	140 (35.7%)
≤42	252 (64.3%)
Simpson's removal grade	
I	137 (34.9%)
II-IV	255 (65.1%)
Pathological grade	
I	362 (92.4%)
II	26 (6.6%)
III	4 (1.0%)
Recurrent	
Yes	26 (6.6%)
No	366 (93.4%)

* *The values in the table are the number of patients, except for age, age in the table presented as the median (IQR). IQR, interquartile range; KPS, Karnofsky performance scale*.

### Univariate Analysis

A chi-square test was used to examine the associations between the clinical characteristics and postoperative recurrence. The results of the univariate analysis of the entire cohort of patients is shown in Table 2. Sex (female vs. male, *P* = 0.001), preoperative KPS (>70 vs. ≤70, *P* = 0.006), tumor size (>42 mm vs. ≤42 mm, *P* = 0.001), tumor shape (regular vs. irregular, *P* = 0.028), peritumoral edema (EI > 4 vs. EI ≤4, *P* = 0.011), tumor surrounding vessels (*P* = 0.032), tumor basal size (>42 mm vs. ≤42 mm, P < 0.001), heterogeneous tumor enhancement (*P* < 0.001), tumor–cortex interface (marked interspace vs. regular border vs. irregular border, *P* < 0.001), brain invasion (*P* < 0.001), Simpson's removal grade (I vs. II-IV, *P* = 0.010), pathological grade (I vs. II-III, *P* < 0.001), and Ki-67 index (≥5% vs. <5%, *P* < 0.001) were identified as prognostic factors for recurrence. There were no significant associations between recurrence and age, tumor location, dural tail sign size (mm) or tumor calcification.

**Table 2:** Univariate analysis of the risk of recurrence.

**Factors**	**Recurrent**	**Non-recurrent**	***P***
Sex			0.001
Female	10(3.7%)	258(96.3%)	
Male	16(12.9%)	108(87.1%)	
Age (y)			0.854
≤60	18(6.8%)	247(93.2%)	
>60	8(6.3%)	119(93.7%)	
Preoperative KPS			0.006
>70	18(5.2%)	327(94.8%)	
≤70	8(17.0%)	39(83.0%)	
Tumor location			0.059
Convexity	10(6.0%)	157(94.0%)	
Skull base	10(8.2%)	112(91.8%)	
Parasagittal sinus	6(11.8%)	45(88.2%)	
Other	0(0.0%)	52(100.0%)	
Tumor size(mm)			0.001
≤42	9(3.6%)	243(96.4%)	
>42	17(12.1%)	123(87.9%)	
Tumor shape			0.028
Regular	14(4.9%)	270(95.1%)	
Irregular	12(11.1%)	96(88.9%)	
Peritumoral edema			0.011
EI ≤4	15(4.9%)	290(95.1%)	
EI>4	11(12.6%)	76(87.4%)	
Dural tail sign size(mm)			0.088
≤10	15(5.3%)	268(94.7%)	
>10	11(10.1%)	98(89.9%)	
Tumor calcification			0.572
NO	22(7.0%)	293(93.0%)	
Yes	4(5.2%)	73(94.8%)	
Tumor-surrounding vessel			0.032
No	15(5.1%)	280(94.9%)	
Yes	11(11.3%)	86(88.7%)	
Tumor basal size(mm)			<0.001
≤42	13(4.2%)	295(95.8%)	
>42	13(15.5%)	71(84.5%)	
Heterogeneous tumor enhancement			<0.001
No	14(4.1%)	324(95.9%)	
Yes	12(22.2%)	41(77.8%)	
Tumor–cortex interface			<0.001
Marked interspace	11(3.7%)	284(96.3%)	
Regular border	7(12.1%)	51(87.9%)	
Irregular border	8(20.5%)	31(79.5%)	
Brain invasion			<0.001
No	11(3.6%)	298(96.4%)	
Yes	15(18.1%)	68(81.9%)	
Simpson's removal grade			0.010
I	3(2.2%)	134(97.8%)	
II-IV	23(9.0%)	232(91.0%)	
Pathological grade			<0.001
I	16(4.4%)	346(95.6%)	
II-III	10(33.3%)	20(66.7%)	
Ki-67 index			<0.001
<5%	8(2.4%)	322(97.6%)	
≥5%	18(29.0%)	44(71.0%)	

*The values in the table are the number of patients. KPS, Karnofsky performance scale. EI, Edema Index*.

### Multivariate Analysis

The independent risk factors for recurrence were identified by a multivariate logistic regression analysis. As shown in Table 3, sex (OR = 2.793, *P* = 0.035), heterogeneous tumor enhancement (OR = 4.452, *P* = 0.002), brain invasion (OR = 2.650, *P* = 0.041), Simpson's removal grade (OR = 5.139, *P* = 0.016), and pathological grade (OR = 3.282, *P* = 0.030) independently predicted recurrence.

**Table 3:** Multivariate analysis to evaluate potential predictive factors for recurrence and the scoring of these factors.

**Factors**	**OR**	**95%CI**	***P***	**Risk score**
Sex				
Female	1			0
Male	2.793	1.076–7.249	0.035	45
Heterogeneous tumor enhancement				
No	1			0
Yes	4.452	1.714–11.559	0.002	65
Brain invasion				
No	1			0
Yes	2.650	1.043–6.733	0.041	42
Simpson's removal grade				
I	1			0
II-IV	5.139	1.355–19.489	0.016	71
Pathological grade				
I	1			0
II-III	3.282	1.123–9.595	0.030	52

*CI, confidence interval; OR, odds ratio*.

### Scoring System

**Figure 2:**
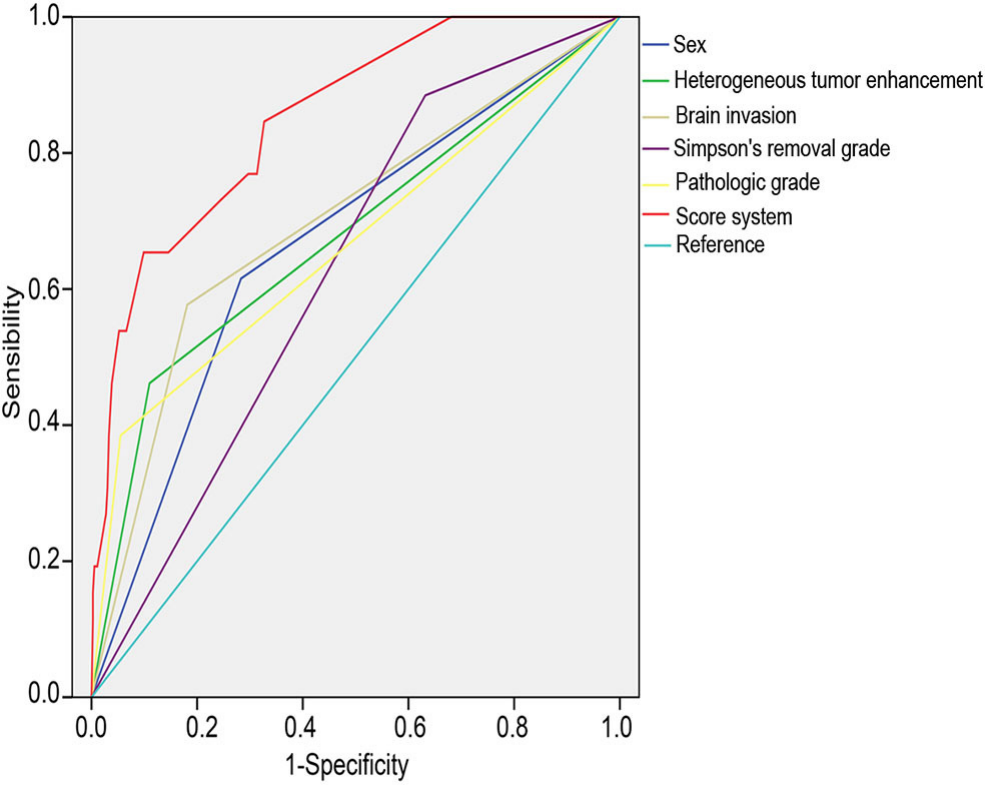
Receiver operating characteristic (ROC) curves of the risk factors. ROC curves evaluating the probability of postsurgical recurrence according to sex, heterogeneous tumor enhancement, brain invasion, Simpson grade, and pathological grade for both individual and combined risk factors.

To establish a scoring system for the accurate prediction of recurrence, we used the independent risk factors identified in the multiple logistic regression analysis. The risk score of each risk factor was calculated by logarithmic transformation and multiplied by 100, resulting in the following risk calculation equation: risk scores = 100^*^log(X), where X = OR (Table 3); these values were summed to determine the composite score. Compared with the individual scores, the composite score improved the accuracy of the prediction of recurrence (i.e., a larger AUC) as follows: combined score, 0.849, 95% CI = 0.776–0.923; sex, 0.660, 95% CI = 0.548–0.772; heterogeneous tumor enhancement, 0.673, 95% CI = 0.551–0.796; brain invasion, 0.696, 95% CI = 0.580–0.911; Simpson's removal grade, 0,625, 95% CI = 0.528–0.722; and pathological grade, 0.665, 95% CI = 0.538–0.792 (Figure 2).

### Development of the Scoring System

**Figure 3:**
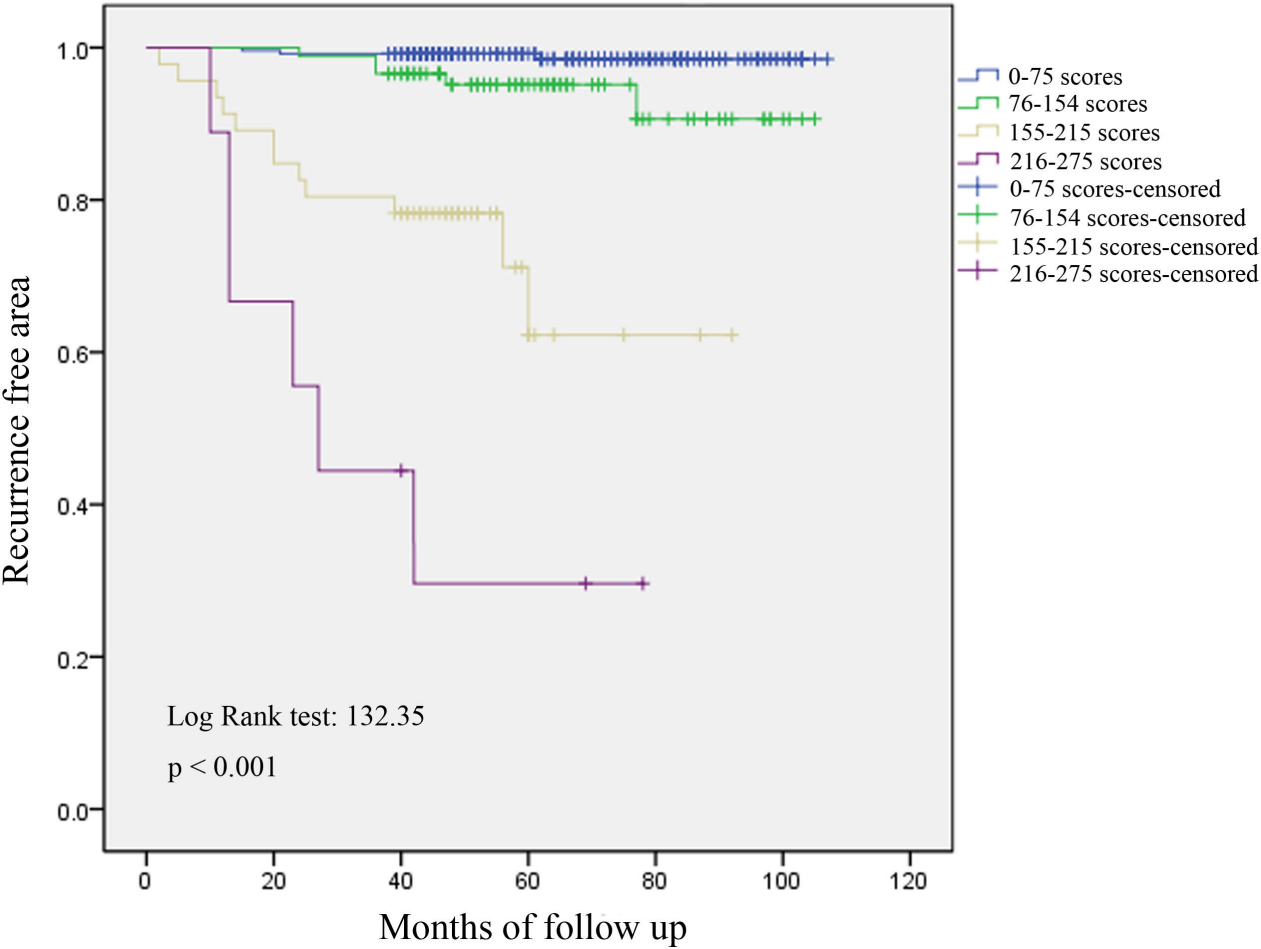
Analysis of recurrence by Kaplan-Meier's curves in four subgroups. Postoperative recurrence differed among the four subgroups, and patients with high scores, especially over 155, had a high risk of postoperative recurrence.

We plotted the ROC curves of the subjects, and the value with the maximal Youden index was selected as the cutoff point for the total scores in each patient; then, the patients was divided into four subgroups (Table 4). The incidences of postoperative recurrence in the patients with scores of 0–75 (*n* = 249), 76–154 (*n* = 88), 155–215 (*n* = 46), and 216–275 (*n* = 9) were 1.2, 5.7, 26.1, and 66.7%, respectively. Consistently, the result of the Kaplan-Meier's curves also showed that postoperative recurrence differed among the four subgroups (Figure 3). The patients with high scores, especially those with scores over 155, had a high risk of postoperative recurrence.

**Table 4:** The postoperative recurrence of different risk groups based on score system.

**Subgroup**	**Score**	**% (fraction) experiencing postoperative recurrence**
Low-risk	0–75	1.2% (3/249)
Medium-risk	76–154	5.7% (5/88)
High-risk	155–215	26.1% (12/46)
	216–275	66.7% (6/9)

*The table showed the percentage of patients experiencing postoperative recurrence based on the score system. Ratios were significantly different between the different risk groups (chi-square tests)*.

## Discussion

Meningiomas are the most common primary brain tumors in adults (13). Currently, the main treatment is surgery, and patients at a high risk of recurrence based on pathological reports or postoperative residuals receive adjuvant radiotherapy. However, identifying the patients who could actually benefit from this approach is controversial. Although several studies have reported the factors associated with postoperative recurrence (3, 14), no comprehensive system is available for the prediction of patients who are a high risk (7, 8) In this study, we combined the clinical, radiological, and pathological characteristics obtained from 392 patients to construct a system for the prediction of postoperative recurrence risk in meningioma. Our data reveal that sex, heterogeneous tumor enhancement, Simpson's removal grade, brain invasion, and pathological grade are independent predictors of meningioma recurrence after surgery. Furthermore, we constructed a new, simple and reliable scoring system and risk-rating model for the prediction of the postoperative recurrence of meningioma.

### Independent Risk Factors and Recurrence

Previous studies have investigated the relationship between sex and recurrence risk. Escribano et al. found that the male sex was an independent risk factor for meningioma recurrence and that men were 2–3 times more likely than women to relapse (15). Wang C's research also revealed that male patients were at a higher risk of recurrence (16). In contrast, in WHO grade II atypical meningioma, Fernandez C et al. showed that the recurrence rates in the females were significantly higher than those in the male patients in their study, which was published in 2016 (17). In our study, we found that males had a higher proportion of WHO grade II-III meningiomas and were more likely to experience recurrence than the female patients. However, the effect of sex on meningioma recurrence remains unclear and may be related to the geographical distribution. A larger cohort study is needed to further investigate this issue.

Advances in radiography technology have made it increasingly important to analyze all radiology results, especially contrast-enhanced MRI, in meningioma. Some previous studies have also described a correlation between heterogeneous tumor enhancement and high-grade meningiomas. Lin et al. reported that heterogeneous enhancement was an independent predictor of high-grade meningioma (5). Durand also observed that all meningiomas with heterogenous enhancement (16/199 cases) were high-grade meningiomas (6). In our study, heterogeneous tumor enhancement was an independent predictor of meningioma recurrence. This finding may indicate local necrosis and higher malignancy in meningioma, which is consistent with previous studies and similar results reported in glioblastoma patients (18).

In the 2016 edition of the WHO Classification of Central Nervous System tumors, brain invasion was added as an independent criterion for atypia meningiomas, which may affect the grading and application of indirect adjuvant therapy. Therefore, this study lays the groundwork for exploring the crucial role of brain invasion, which can influence considerations regarding meningioma patients' postoperative treatment and prognosis (19). Many recent studies have also reported that a correlation exists between brain invasion and prognosis or recurrence (20–25), and our study confirms this finding. We found that brain invasion, Simpson's removal grade and the pathological grade were independent predictors of meningioma recurrence. To date, several studies have suggested that the most important prognostic factor for tumor recurrence is the histological grade (26–28). While focusing on grade I tumors, Marciscano et al. (26) demonstrated that a relationship exists between the recurrence rate and histological grade as follows: in WHO grade I tumors, the chance of recurrence was 7–25%; in grade II, the chance of recurrence was 29–59%; and in grade III, the chance of recurrence was 60–94%. Additionally, some studies have indicated Simpson's removal grade is closely correlated with the risk of recurrence (29, 30). Aizer et al. reviewed 575 and 64 patients diagnosed with atypical and malignant meningioma, respectively, and assessed the adjusted impact of gross total resection (GTR) and subtotal resection (STR) on all-cause mortality. The results showed that the extent of resection was an important index for predicting the prognosis of patients with atypical and malignant meningiomas (31). Currently, in clinical practice, Simpson's removal grade and the pathological grade are the parameters most commonly used by neurosurgeons to assess postoperative recurrence. Some studies found that the Ki-67 index was an important risk factor; however, the Ki-67 index was not an independent risk factor in our study. We believe that there may have been interference between the variables.

### Prediction Model

According to the results of the univariate and multivariate analyses, we constructed a predictive scoring system and risk-rating model of meningioma recurrence. As shown in Figure 2, the AUC of the combined scores was significantly higher than that of the individual scores, indicating that the model was able to screen patients with a high risk of recurrence. Based on the scoring system, the patients were divided into four subgroups with scores of 0–75, 76–154, 155–215, and 216–275. According to the recurrent curves shown in Figure 3 and the incidences of postoperative recurrence, we consider that the subgroup scoring 0–75 is at a low risk level, and postoperative adjuvant therapy is not required; the subgroup scoring 76–154 is at a medium risk level, and postoperative adjuvant therapy should be determined based on the clinical features and follow-up; the subgroups scoring 155–215 and 216–275 are at a high risk level, and further adjuvant therapy is recommended after surgery. Therefore, this model could help optimize the treatment strategies and the adoption of comprehensive adjuvant therapy in high-risk patients with the aim to decrease or slow tumor recurrence.

In a recent study, Escribano et al. reviewed 125 patients with parasagittal meningiomas and constructed a binary logistic regression model. These authors concluded that the male sex, tumor size and histologic type were independent risk factors for recurrence (15). Chohan and colleagues retrospectively analyzed the clinical data of 60 patients with histologic atypia/anaplasia at the time of the first recurrence of meningioma. A competitive risk regression model was used to analyze the predictors of second recurrence. These authors suggested increasing radiation therapy to better control the tumor and challenged the importance of the extent of resection in the first recurrence (32). Both above-described studies built a model and predicted the recurrence of meningiomas; however, compared to our study, these two previous studies included fewer patients, focused only on the risk factors and did not further quantify or classify the results. In 2014, Domingues and colleagues conducted a comprehensive and in-depth analysis of 302 meningioma patients. These authors included clinical, imaging, pathology and results and genetic testing and were the first to model, quantify and build a new prognostic classification for meningioma patients. Although these authors provided different strategies for the treatment of meningioma (33), in clinical practice, it is not easy to obtain all the information used in their study, especially the data of whole exome sequencing (WES) of the tumor tissue. In our study, routinely collected data ***were***used, rendering our approach more accessible, more practical and easier to implement and promote in clinical practice with high accuracy.

### Limitations

Our study has some limitations. First, the sample size was not large, and the level of loss to follow-up was relatively high, which may have led to statistical bias. Furthermore, this study was a retrospective study performed in a single institution, and the follow-up duration was not long enough for benign tumors. Therefore, it is necessary to conduct a large-scale multicenter study to further validate our scoring system before it can be used in daily practice.

## Conclusions

In our study, we identified the independent risk factors for postoperative recurrence of meningioma and constructed a scoring system for recurrence. This scoring system is a simple and reliable instrument that can be used to identify meningioma patients at risk of postoperative recurrence and could help optimize individualized treatment in a clinical setting, especially for high-risk patients.

## Data Availability Statement

The raw data supporting the conclusions of this article will be made available by the authors, without undue reservation.

## Ethics Statement

This study was approved by the Clinical Research Ethics Committee of the First Affiliated Hospital of Wenzhou Medical University. Given the retrospective nature of the study, patient informed consent was waived.

## Author Contributions

ZS and WZ: conception and design, approved the final version of the manuscript on behalf of all authors, administrative/technical/material support, and study supervision. ZZ, CW, JX, CW, LX, QL, JL, and LC: acquisition of the data. ZS, ZZ, CW, JX, QL, JL, LC, and LX: analysis and interpretation of the data. ZS, WZ, and ZZ: drafting of the article. All authors: critical revision of the article, reviewed submitted version of the manuscript.

## Conflict of Interest

The authors declare that the research was conducted in the absence of any commercial or financial relationships that could be construed as a potential conflict of interest.
